# The Effects of Vegetarian and Vegan Diets on Gut Microbiota

**DOI:** 10.3389/fnut.2019.00047

**Published:** 2019-04-17

**Authors:** Aleksandra Tomova, Igor Bukovsky, Emilie Rembert, Willy Yonas, Jihad Alwarith, Neal D. Barnard, Hana Kahleova

**Affiliations:** ^1^Faculty of Medicine, Institute of Physiology, Comenius University in Bratislava, Bratislava, Slovakia; ^2^Center for Clinical Nutrition, Bratislava, Slovakia; ^3^Physicians Committee for Responsible Medicine, Washington, DC, United States; ^4^Adjunct Faculty, George Washington University School of Medicine and Health Sciences, Washington, DC, United States

**Keywords:** gut microbiota, fiber, nutrition, plant-based diet, postbiotics

## Abstract

The difference in gut microbiota composition between individuals following vegan or vegetarian diets and those following omnivorous diets is well documented. A plant-based diet appears to be beneficial for human health by promoting the development of more diverse and stable microbial systems. Additionally, vegans and vegetarians have significantly higher counts of certain *Bacteroidetes*-related operational taxonomic units compared to omnivores. Fibers (that is, non-digestible carbohydrates, found exclusively in plants) most consistently increase lactic acid bacteria, such as *Ruminococcus, E. rectale*, and *Roseburia*, and reduce *Clostridium* and *Enterococcus* species. Polyphenols, also abundant in plant foods, increase *Bifidobacterium* and *Lactobacillus*, which provide anti-pathogenic and anti-inflammatory effects and cardiovascular protection. High fiber intake also encourages the growth of species that ferment fiber into metabolites as short-chain fatty acids (SCFAs), including acetate, propionate, and butyrate. The positive health effects of SCFAs are myriad, including improved immunity against pathogens, blood–brain barrier integrity, provision of energy substrates, and regulation of critical functions of the intestine. In conclusion, the available literature suggests that a vegetarian/vegan diet is effective in promoting a diverse ecosystem of beneficial bacteria to support both human gut microbiome and overall health. This review will focus on effects of different diets and nutrient contents, particularly plant-based diets, on the gut microbiota composition and production of microbial metabolites affecting the host health.

## Introduction

Recent studies of the human microbiome have emerged as an area of popular interest. For decades, many investigations have elucidated the impact of the human gut microbiota on the physiology of the host, with new and unexpectedly broad implications for health and disease.

The human microbiota, defined as the total of all microbial taxa associated with human beings (bacteria, viruses, fungi, protozoa, archaea), consists of a newly estimated 3 × 10^13^ (trillion) microbes harbored by each person (1). The term *microbiome* is often incorrectly used interchangeably with the term microbiota. However, microbiome refers to the catalog of these microbes and their genes. The human gut microbiome represents ~3.3 million non-redundant microbial genes, which outnumbers the human genome of some 21,000 genes in the ratio of ~150:1 (2). Interestingly, the diversity among the microbiomes of two different individuals is vast compared to their human genomic variation; humans are about 99.9% identical to each other in terms of their genome (3), but their gut microbiome can be up to 80–90% different (4).

Recent advancements in laboratory techniques have revealed functions of the human gut microbiota related to immunity and the gastrointestinal, brain, and cardiovascular systems. Research has also suggested a profound effect of the human gut microbiota on host cells and genes. This extensive interaction has suggested that the microbiome functions effectively as a separate “organ.”

Several studies have suggested that there are three basic bacterial enterotypes (5) (1) genus *Prevotella* (considered to be mostly anti-inflammatory and otherwise protective), (2) genus *Bacteroides* (more pro-inflammatory and possibly related to the heightened risk of metabolic syndrome and other pathological conditions), and (3) genus *Ruminococcus* (of which the biological significance is less evident) (6).

An imbalance of the gut microbiota has been linked with gastrointestinal conditions such as reflux, peptic ulcers, irritable bowel syndrome, non-alcoholic steatohepatitis, and inflammatory bowel disease. Additionally, some systemic conditions such as obesity, atherosclerosis, type 2 diabetes, cancer, Alzheimer's and Parkinson's disease, amyotrophic lateral sclerosis, autism spectrum disorder, atopy etc., also appear to be linked to unfavorable changes in gut microbiota composition (7–17). An accumulating body of evidence points to the gut microbiota as a mediator of dietary impact on the host metabolic status. Current research is focusing on the establishment of causal relationships in people and the development of therapeutic interventions such as personalized nutrition (18).

Dietary composition appears to have long-term and acute effects on the gut microbiota ecosystem (19, 20). Different long-term dietary patterns, such as vegetarian/vegan vs. omnivorous diets, have significant influence on gut microbiota composition. The different gut microbiota content is shown to provide different food nutrients metabolites, termed postbiotics. For instance, SCFAs, phytoestrogens, or isothiocyanates are more linked with the plant-based food, while TMAO and secondary bile acids with the meat-based diet. These and other postbiotics take part in the metabolism of the host in different ways. This review will focus on some general as well as specific aspects of this dynamic field of research.

## Gut Microbiota: General Aspects

In addition to bacteria, the gut is host to multiple kingdoms: archaea, viruses, and eukaryotes, including fungal species. The gut microbiota is represented by more than 1,000 microbial species, belonging primary to just two phyla: *Bacteroidetes* and *Firmicutes* (21). Based on human stool samples, overall, the genera *Bacteroides, Prevotella, Bifidobacterium, Eubacterium, Clostridium, Streptococcus*, and *Enterobacteriaceae* are most commonly found. It should be noted that stool samples provide reasonable estimations of the gut microbiota rather than a complete representation (22). This is because anaerobic species often attach to the gut mucosa, making it difficult to identify all bacterial species present in the large intestine. Also, it is probable that the biological significance of any genera or species is not given by its relative proportion in the whole ecosystem. Rather, its significance is observed through its metabolism/postbiotics and effects on the enteric nervous system, local immunity, brain, and genes.

## Effect of Diet on Gut Microbiota Composition

The difference in gut microbiota composition between individuals consuming a vegan/vegetarian and an omnivorous diet is well documented. Research shows that vegetarian/vegan diets foster different microbiota when compared to omnivores, with only a marginal difference between vegans and vegetarians (23). Changes in microbiota composition might be due to differences in bacteria directly consumed through food, differences in substrates consumed, variations in transit time through the gastrointestinal system, pH, host secretion influenced by dietary patterns, and regulation of gene expression of the host himself and/or his/her microbiota (24).

A plant-based diet appears to be beneficial for human health by promoting the development of a more diverse gut microbial system, or even distribution of different species (25, 26).

### Diversity and Richness of the Gut Microbiota

The diversity of the microbiota appears to have an important association with BMI, obesity, and arterial compliance; and a majority of the research suggests that a plant-based diet fosters a greater microbial diversity. Klimenko et al. found a positive association between alpha-diversity, or local microbial richness, and long-term fruit and vegetable intake (*p* < 0.05) (27). Likewise, Martinez et al. observed that adding whole-grain barley, brown rice, or a mixture of the first two to the diet of volunteers resulted in an increase in microbial diversity (*n* = 28) (28). Klimenko et al. also found a negative association between alpha-diversity and BMI (*p* < 0.05) (27).

However, a short-term dietary intervention advising increased fiber consumption resulted in a slight but significant decrease in diversity (*p* < 0.001). The researchers suggest this reduction in diversity might be the result of a rapid dietary change resulting in a temporary disruption to the microbial composition. This hypothesis of transitory microbial “stress” also explains the slight but significant increase in *Enterobacteriaceae* as a result of the intervention (*p* < 0.05). *Enterobacteriaceae* abundance is typically lower on a vegan diet vs. an omnivorous one (*P* < 0.05) (29). This is likely due to the greater presence of butyrate-producing bacteria on a higher fiber diet, which can lower colonic pH, preventing the growth of pathogenic bacteria, such as *Enterobacteriaceae* (30).

Verdam et al. observed reduced microbial diversity in obese vs. non-obese study participants (*n* = 28). The obese participants also displayed a reduction in the *Bacteriodetes*:*Firmicutes* ratio and an increase in *Proteobacteria*, a pro-inflammatory phylum. Likewise, an increase in C-reactive protein was observed (*p* < 0.001) which inversely correlated with the *Bacteriodetes*:*Firmicutes* ratio (*p* < 0.05). These observations suggest a pro-inflammatory effect of obesity-related microbiota (31). On the other hand, participants from the Adventist Health Study-2 (60,903) following a vegan diet displayed the lowest BMI values when compared with those following a vegetarian or omnivorous diet (32). These findings indicate that a vegan diet, associated with lower body weight, might benefit microbial diversity and protect against inflammation.

Menni et al. observed that carotid-femoral pulse wave velocity, a measure of arterial stiffness, was negatively associated with microbiome diversity (*p* = 0.001) in women (*n* = 617) (33). This correlation remained significant after adjusting for insulin resistance and visceral fat. Arterial stiffness is oftentimes caused by hyperglycemia or hyperinsulinemia (34) and is significantly correlated with inflammatory adipokine levels. The researchers suggest the association between arterial stiffness and microbial diversity can be explained partially by the role of the gut in modulating systemic inflammation. Thus, an increase in microbial diversity might improve systemic inflammation, thereby reducing arterial stiffness.

Additionally, vegans and vegetarians have a significantly greater richness (alpha diversity) compared to omnivores, specifically counts of certain *Bacteroidetes*-related operational taxonomic units (OTUs) (35). It seems likely that many health benefits of vegetarian/vegan diets are, in part, mediated by the gut microbiota—not only through the higher relative abundance of those OTUs that are currently considered to be protective (*Bacteroidetes, Prevotella, Roseburia*, etc.), but also from postbiotic and epigenetic effects on various risk factors for chronic inflammation and chronic degenerative diseases (36).

### Effects of Diet on the Bacteroidetes:Firmicutes Ratio

Despite significant inter-individual differences, a healthy adult intestinal microbiome is characterized by the dominance of these *Bacteroidetes*-related OTUs along with those of the *Firmicutes* phylum (37, 38). Research has shown variability in these phyla concentrations to be heavily affected by diet, specifically the ratio between the two when comparing omnivorous diets of the type common in North America, vs. a vegetarian/vegan diet. One study compared the bacterial composition between Indian (*n* = 11) and Chinese (*n* = 5) adults (39). While both populations ate diets centered around carbohydrates and vegetables, the Chinese diet was heavier in animal fat and protein than the Indian diet of whole grains and plant-based vegetarian foods. The percentage of *Bacteroidetes* within the microbiomes of Indian participants was nearly four times greater than in the Chinese, 16.39% vs. 4.27%, respectively (*p* = 0.001). The higher abundance of *Bacteroidetes* in Indians was hypothesized to be due to their lower consumption of animal products; indicating a diet lower in animal products to be associated with greater *Bacteroidetes* counts.

Another study compared the fecal microbiota of Italian children (*n* = 15) vs. the fecal microbiota of children living in a rural western Africa, specifically in Burkina Faso (*n* = 14) (40). The Italian children typically consumed a Western diet, high in animal protein, sugar, starch, and fat and low in fiber. The African children of Burkina Faso consumed a diet low in fat and animal protein and rich in starch, fiber, and plant protein. The abundance of *Firmicutes* was twice as much in the Italian children than in the Burkina Faso children (63.7 vs. 27.3%, respectively). The abundance of *Bacteroidetes* in the Italian children was less than half of that seen than in the Burkina Faso children (22.4 vs. 57.7%, respectively). A decrease in *Firmicutes* levels, usually occurring in favor of *Bacteroidetes* and *Bifidobacteria*, as seen in response to an increase in resistant starches, may be beneficial in preventing and treating obesity (41). While these correlations between diet and microbiota composition are observed among different populations, it is important to consider other factors that may play a role, such as ethnicity, host genotypes, environmental factors, etc.

Research has shown that the balance of *Bacteroidetes* and *Firmicutes* is an important marker for obesity and higher BMI. Specifically, a decreased *Bacteroidetes*:*Firmicutes* ratio has a strong negative correlation with BMI (r_s_ = 0.59, *P* < 0.001) (31). A possible explanation for this correlation may be found in the observation that a 20% increase in *Firmicutes* and a corresponding decrease in *Bacteroidetes* abundance is associated with a 150 kcal/day increase in energy harvest, resulting in weight gain overtime. Therefore, an increased *Bacteroidetes*:*Firmicutes* ratio, as seen on a high fiber, plant-based diet, may result in weight loss by reducing the amount of energy extracted from the diet. Further research is needed to determine whether the increase in energy harvest due is due to the *Bacteroidetes*:*Firmicutes* ratio promoting adiposity or representing a host-mediated adaptive response to limit energy uptake (42).

Studies have also shown opposite trends in *Firmicutes* and *Bacteroidetes*. One study compared US children eating a Western diet to Bangladeshi children consuming a plant-based diet of rice, bread, and lentils. The *Bacteroidetes*:*Firmicutes* ratio was three times higher in the US children consuming the Western diet (43). This opposes the previous prediction of a Western diet resulting in a decreased *Bacteroidetes*:*Firmicutes* ratio. The researchers noted age and geographical differences as potential explanation for this departure from the expected ratio, as well as inter-subject variability. Another study asked participants to increase their fiber consumption and avoid Western diet foods. While prior studies would have suggested an increase in *Bacteroidetes*:*Firmicutes*, the ratio decreased (0.13 ± 0.2 to 0.03 ± 0.09, Wilcoxon paired test *p* < 0.0001, *n* = 430) (27). Another study analyzed the microbial composition of lean and obese subjects (*n* = 98) and observed that, when compared to lean subjects, overweight and obese volunteers presented a higher ratio *Bacteroidetes* to *Firmicutes* (*P* = 0.001 and *P* = 0.005, respectively) (44). Likewise, comparison of bacterial phyla did not show a significant difference in the *Bacteroidetes* to *Firmicutes* ratio between obese and lean volunteers (*n* = 20) (45). These examples reflect the difficulties in broadly linking certain phyla to particular diets. The primary challenge in analyzing specific microbiota is the need to consider the state and interaction dynamic of microbes encompassing the whole microbiome.

### Effects of Diet on Enterotypes

As mentioned above, there are three main enterotypes observed in human microbiomes: *Prevotella, Bacteroides, and Ruminococcus*. *Prevotella*, a genus of the *Bacteroidetes* phyla, appears to be significantly richer in response to a vegan diet. In the previously mentioned study by De Filippo et al., *Prevotella* was exclusively present in the children of Burkina Faso consuming a diet low in fat and animal protein and rich in starch, fiber, and plant protein when compared to children living in Italy consuming a Western diet, high in animal protein, sugar, starch, and fat, and low in fiber (40). Another study compared the diets of 178 elderly residents living in either the community or in long-term residential care (46). The community group was found to consume a low to medium fat, high fiber diet; while the residents in long-term care consumed a moderate to high in fat, and low fiber diet. The study found that those in the community, eating a profile more reflective of a plant-based diet, more frequently had gut communities of the *Prevotella* enterotype.

The study comparing Indian and Chinese adults shows similar results (39). As expected, the Indians who were consuming less animal products and more plant-based foods than the Chinese had a significantly greater percentage of *Prevotella* (13.07 vs. 0.58%, respectively). When the abundance of *Prevotella* was analyzed in Thai vegetarians vs. non-vegetarians, the vegetarians were found to have significantly higher numbers of *Prevotella* (*p* = 0.005) (47). Other studies have shown vegan/vegetarian diets, high in plant-based foods, to be associated with high abundances of *Prevotella* (48, 49). This suggests additional support for greater *Prevotella* presence in those whom consume less animal products and more plant-based food. While mice studies suggest *Prevotella* to improve glucose metabolism by improving glycogen storage (50), the current lack of any additional research makes *Prevotella* merely a genus to describe an overall ecosystem of human gut bacteria, primarily under a plant-based diet.

*Bacteroides*, another main enterotype and genus of the *Bacteroidetes* phyla, also appears to be affected by diet but in a different way to *Prevotella*. *Bacteroides* has been positively correlated with long-term diets rich in animal protein and saturated fat (20, 27). This is likely due to their ability to tolerate bile, which is common in gut environments of those who consume animal products. In the study mentioned earlier comparing children in the US eating a Western diet vs. children in Bangladesh consuming a plant based diet, *Bacteroides* was the major genus in the US children's microbiota. High proportions of *Bacteroides* are found in the gut of humans consuming a Western diet and the opposite is found in those consuming a high fiber diet of fruits and legumes (27, 37, 43, 47, 48).

*Ruminococcus* is the third major enterotype and is associated with long term fruit and vegetable consumption. Species of this genus are specialized in degrading complex carbohydrates, such as cellulose and resistant starch, found in plant based foods (51). These microbes degrade dietary fibers, producing butyrate, which acts as an anti-inflammatory compound. *Ruminococcus* is positively associated with low BMI and negatively associated with poor lipid profile (27). Likewise, abundance of *Rumminococcus* has been linked to lower endotoxemia and lower arterial stiffness, a predictor of cardiovascular risk (33). Walnut consumption has been significantly associated with enrichment of *Ruminococcus* as well (38). However, *Ruminococcus* has also been linked to low dietary fiber consumption in college students. While these microbes degrade complex carbohydrates, they also break down the resistant starches found in refined grain products (52). *Ruminococcus* might also play a role in the conversion of animal-derived choline to trimethylamine (TMA) (53). Thus, the abundance of *Ruminococcus* is influenced by both animal and plant based diets.

### Effects of Diet on Additional Bacteria

While *Bacteroides* can be pro-inflammatory and their concentration is associated with long term consumption of animal products, a study analyzing 11 vegetarians, 20 vegans, and 29 omnivores (49) found a discrepancy in this generalization. In addition to finding *Clostridium clostridioforme* and *Faecalibacterium prausnitzii*, both considered to be health protective, in higher relative abundance in the vegetarians/vegans compared to the omnivores, *Bacteroides thetaiotaomicron* was also observed in higher abundance in these groups. This discrepancy in categorizing bacteria abundance under a plant-based diet vs. animal-based diet is not uncommon. *Clostridium* cluster XIVa was found in lower ratio in the vegetarian/vegans, contrary to a study showing *Clostridium* cluster XIVa bacteria to be a major component of gut microbiota in vegetarian women (103). Therefore, while generalizations can be made, some genus subtypes will be outliers. This discrepancy in some bacterial phyla in response to diet has been acknowledged by previous review papers and has been attributed to various reasons, such as different microbiome profiling methodologies, different host genetics, body mass index, and red wine and aspartame consumption (54, 55). These are all factors that have been shown to possibly modify our microbiota. Therefore, further studies are warranted in order to isolate their effects from those due to a plant based vs. omnivorous diet.

Taken together, dietary habits influence the composition of the intestinal microbiota. While dietary changes have a relatively fast impact (51) (within a week) on the microbial composition and consequently on its metabolites, these effects are modest and reversible (24). For example, changes of microbiota and immune parameters after a 3-month vegetarian diet are significant, but do not reflect the degree of change that occur with a long-term vegetarian diet (56).

## How Plant Food Components Influence Gut Microbiota.

### Nutrient Bioavailability

Consuming food nutrients with low bioavailability has recently been found to be important. Lower nutrient bioavailability, found in larger food particles, intact plant cell walls, and food without thermal treatment, means that more nutrients reach lower in the gastrointestinal system, thus enriching nutrient delivery to the gut microbiota (57). This helps support normal gut microbiota development and function (57). Modern westernized diets contain more ultra-processed foods and acellular nutrients, or nutrients not containing cells. These components are more easily absorbed in the small intestine, depriving the colon of important nutrients, which may alter the composition and metabolism of the gut microbiota (58). Acellular food, e.g., sugar, has been shown to induce inflammation in young infants, adolescents, women of child-bearing age, and older adults. Whole plant foods have protective effects, favoring the growth of beneficial fiber-degrading bacteria in the colon (58).

### Carbohydrates

Unlike digestible carbohydrates, non-digestible carbohydrates, such as resistance starch, and some sugars, reach the large intestine where they can be fermented by the gut microbiota to provide energy or produce postbiotics. However, both digestible and non-digestible carbohydrates may influence the gut microbiota. Digestible carbohydrates from fruits (e.g., glucose, sucrose, and fructose) have been shown to reduce *Bacteroides* and *Clostridia* (54). Non-digestible carbohydrates most consistently increase lactic acid bacteria, *Ruminococcus, E. rectale*, and *Roseburia*, and reduce *Clostridium* and *Enterococcus* species (54). Both digestible and non-digestible carbohydrates have been shown to increase *Bifidobacteria*, genus of the *Actinobacteria* phylum.

A study compared the *Bifidobacteria* levels in response to a randomized, double-blind, crossover trail. Participants consumed both a standard enteral formula and a formula supplemented with fructooligiosaccharides (FOS) and fiber (59) as a sole source of nutrition for 14 days. FOS and fiber are both forms of carbohydrates found naturally and abundantly in plant foods–bananas, artichokes, onion, etc. While the volume of formula prescribed was based on individual energy expenditures, a benchmark of 2,000 calories of the FOS/fiber formula provided 10.2 g of FOS and 17.8 g of fiber. The average daily intake of fermentable non-digestible carbohydrates is estimated to be 10 g from inulin and FOS (60). This amount does not include meals and products supplemented with inulin and FOS, which typically add an additional 3–10 g/portion. Therefore, 10.2 g of FOS in the formula is realistic for human consumption. 17.8 g of fiber in the formula is also realistic for human consumption as the average US male and female intake is 18 g and 15 g, respectively ^1^.

*Bifidobacterium* is a butyrate-producing genus known to play a protective role in the human gut barrier by providing defense against pathogens and diseases. When participants were given formulas with FOS and fiber, their *Bifidobacteria* increased from 5.1 to 26.6% (*P* = 0.003) after 14 days. When formula was given without FOS and fiber, *Bifidobacteria* only increased from 3.3 to 8.6% (*P* = 0.073). A negative correlation between baseline *Bifidobacteria* and magnitude of the bifidogenic effect was observed, indicating that those with lower initial amounts of *Bifidobacteria* benefit most from fructooligiosaccharides and fiber intake. In contrast, high consumption of cholesterol from animal products, was strongly associated with a lower abundance of *Bifidobacteria* (adj. *p* = 0.008).

While these studies suggest that *Bifidobacterium* increase in response to a fiber-rich, high carbohydrate diet, other studies have shown conflicting results. One important confounding factor may be alcohol intake, which has been strongly associated with a lower abundance of *Bifidobacteria* (adj. *p* = 0.006). Researchers comparing *Bifidobacteria* levels in vegans, vegetarians, and controls, found *Bifidobacteria* to be significantly lower (*p* = 0.002) in vegan samples than in controls eating a standard omnivorous diet. No difference between vegans and vegetarians was observed (29). Another study observed higher *Bifidobacteria* levels in meat eaters compared to participants who switched to a vegetarian diet for 4 weeks after eating a mixed Western diet, high in fat and meat (58). The relative decrease of *Bifidobacterium* in vegetarians and vegans may be explained by a relative abundance of other protective bacteria species, such as *Prevotella*. *Prevotella* has been observed confers anti-inflammatory effects (40) and can decrease the growth of other bacteria by competing for fiber as an energy substrate (61).

A recent *in vitro* study elucidated the specific mechanism of action of carbohydrates, specifically selected dietary fibers, on gut microbiota. The study found the following fibers to have differing prebiotic effects: inulin, alpha-linked galacto-oligosaccharides, beta-linked galacto-oligosaccharides, xylo-oligosaccharides from corn cobs and high-fiber sugar cane, and beta-glucan from oats (62). Beta-glucan induced the growth of *Prevotella* and *Roseburia* with a concomitant increase in SCFA propionate production. Inulin and all oligosaccharides had a strong bifidogenic effect (62). This study also showed that all natural sugars, most notably non-digestible forms like inulin and oligosaccharides, increase SCFA levels (62). The prebiotic effects differ due to the type of bacteria that each fiber is broken down by. This is determined through bacterial specificity in which specific gene clusters within the bacterial genome dictate the saccharolytic enzymes that the bacteria can produce and, therefore, whether they can metabolize the prebiotic substrate (63). Non-digestible carbohydrates not only act as prebiotics by promoting the growth of beneficial microorganisms, but also reduce proinflammatory cytokine production, concentrations of serum triglycerides, total cholesterol, and LDL-cholesterol (54). Thus, non-digestible carbohydrates might confer protective effects against cardiovascular disease and central nervous system disorders.

### Proteins

A majority of the studies have noted that protein consumption correlates positively with microbial diversity (54). However, animal and plant-proteins influence the gut microbiota in different ways. For instance, individuals consuming a high animal protein diet, from beef which is also high in fat, displayed lower abundances of bacteria, such as *Roseburia, Eubacterium rectale*, and *Ruminococcus bromii*, that metabolize dietary plant polysaccharides (51). Populations of bacteria that increase in response to a high animal protein diet when compared to subjects consuming a meatless diet are typically bile-tolerant microorganisms, such as *Bacteroides* and *Clostridia* (64). Additionally, a high-protein diet typically limits carbohydrate intake, which may lead to a decrease in butyrate-producing bacteria, and thereby to a proinflammatory state and an increased risk of colorectal cancer (65).

Individuals consuming pea protein exhibit increases in beneficial *Bifidobacterium* and *Lactobacillus* and decreases in pathogenic *Bacteroides fragilis* and *Clostridium perfringens* and, consequently increases in intestinal SCFA levels (54). Likewise, plant-derived proteins have been associated with lower mortality than animal-derived proteins (54).

### Fats

Current evidence suggests that both the quantity and the quality of consumed fat significantly impact the gut microbiota composition (65).

A plant-based diet is generally naturally low in fat, which favors beneficial *Bifidobacteria* in human gut microbiota. The fat that does come from a vegan/vegetarian diet is made up of predominantly mono and polyunsaturated fats, which increase the *Bacteroidetes*:*Firmicutes* ratio, and on the genera level, increase lactic acid bacteria, *Bifidobacteria* and *Akkermansia muciniphila* (54). Nuts, particularly walnuts, have been shown to improve the gut microbiota by increasing *Ruminococcaceae* and *Bifidobacteria*, and decreasing *Clostridium* sp. cluster XIVa species (38).

On the other hand, saturated fat, found almost exclusively in animal sources, increases *Bilophila* and *Faecalibacterium prausnitzii*, and decreases *Bifidobacterium* (54). Some studies report that this change activates inflammation (induces pro-inflammatory cytokines such as IL-1, IL-6, and TNF-α) and leads to metabolic disorders (66). High consumption of saturated and trans fat, predominately found in a Western diet, increases the risk of cardiovascular disease and reduces *Bacteroidetes, Bacteroides, Prevotella, Lactobacillus* ssp. and *Bifidobacterium* spp, and increases *Firmicutes* (40, 67).

N-3 polyunsaturated fatty acids, have been found to result in either no change to the microbiota, or beneficial increases in *Bifidobacterium, Adlercreutzia, Lactobacillus, Streptococcus, Desulfovibrio*, and *Verrucomicrobia* (*Akkermansia muciniphila*) (54, 67).

### Polyphenols

Polyphenols, or naturally occurring plant metabolites (68), in plant foods increase *Bifidobacterium* and *Lactobacillus* abundance, which provide anti-pathogenic and anti-inflammatory effects and cardiovascular protection (54). Common polyphenol-rich foods include fruits, seeds, vegetables, tea, cocoa products, and wine. For example, polyphenol extracts from tea generate an increase in *Bifidobacterium* and *Lactobacillus–Enterococcus* spp., which then yields an increased SCFA production on human microbiota *in vitro* (69).

## Influence of Microbiome Postbiotics on Human Health

Research on the gut-brain, gut-lung, and gut-liver axes highlights the importance of the microbiome on systemic human health. Studies note changes in central neural chemistry, inflammatory lung conditions, and non-alcoholic fatty liver disease pathogenesis with changes to microbial composition (70–72). The mechanism of communication among these organs stems from the microbial products and microbial metabolites of ingested nutrients. These products can be diet-independent (such as lipopolysaccharides, ribosomally synthesized post-translationally modified peptides etc.), but here we would like to describe a few examples of well-known diet-dependent metabolites, such as SCFA and others. Depending on the bacteria and location along the intestinal tract, different bioactive molecules can be produced from different prebiotics and nutrients (70, 73). Microbial metabolites can have diverse positive health effects including local anti-inflammatory and immunomodulatory effects, and systemic anti-obesogenic, antihypertensive, hypocholesterolemic, anti-proliferative, and antioxidant effects (74). These postbiotic effects result from modulation of gene expression, metabolism, and intestinal functioning and depend on microbiota composition and substrates, largely dependent on diet.

### Short-Chain Fatty Acids

SCFAs act as a substrate to maintain colonic epithelium, and are correlated with plant based food consumption (56). Maintenance of the intestinal barrier prevents endotoxemia and the subsequent inflammatory effects (75, 76). SCFAs acetate, propionate, and butyrate, are mostly microbial metabolites of fermented fiber and other carbohydrates, although a small fraction can derive from proteins. The fecal levels of these metabolites (and the corresponding esters) positively correlate with the consumption of fruits, vegetables, and legumes. Thus, their levels significantly increase in people who begin a plant-based diet. Interestingly, an increase in SCFAs is observed when omnivores consume a Mediterranean diet rich in fruit, legumes and vegetables (77).

While specific gut microbes are predisposed for SCFA production, different bacteria are known to produce different SCFAs. For example, enteric bacteria, such as *Akkermansia muciniphila, Bifidobacterium* spp., *Prevotella* spp., and *Bacteroides* spp. produce acetate; *Bacteroides* spp. produce propionate; and *Coprococcus* produces butyrate (78). The most butyrate producing bacteria are in *Clostridium* Cluster XIVa, IV, and XVI. These species are positively correlated with consumption of plant foods, and produce SCFAs that yield several health benefits.

The protective role of acetate, propionate and butyrate against different types of disease, such as type 2 diabetes, inflammatory bowel disease, and immune diseases, is well documented. For example, it has been shown that SCFAs promote immunity against pathogens (78), and are important components for microglia function and maturation and control of the blood–brain barrier integrity (79). Other effect of SCFAs is to increase thermogenesis, preventing/treating obesity (80, 81). SCFAs serve as energy substrates for colonocytes, as well as for the body generally. For example, propionate serves as a gluconeogenic substrate in the liver and in the intestine (78).

Microbial interactions with dietary polysaccharides and the resulting SCFAs are important energy and signaling molecules. It is becoming increasingly accepted that butyrate-producing bacteria and butyrate, *per se*, may be beneficial for human health (78). Butyrate has been shown to play a key role in gut physiology as a major carbon source for colonocytes. It helps regulate critical functions of the intestine, such as intestinal motility, mucus production, visceral sensitivity, the epithelial barrier, immune homeostasis, and the mucosal oxygen gradient (82, 83). Thus, dietary fiber and carbohydrates can affect SCFA degradation while altering the abundance of the associated microbes. Taking together, diets rich in fiber might provide benefits to the intestine, as well as overall health.

### Phytoestrogens

Phytoestrogens are plant-derived polyphenols that interact with estrogen receptors with either agonist or antagonist actions. A large majority of polyphenols are delivered to the gut, given their 1% bioavailability (57). The protective effects of plant polyphenols, particularly their anti-cancer, anti-inflammatory, and antioxidant effects, and their association with decreased risks of cardiovascular disease, obesity, diabetes, osteoporosis, and amyloid formation have been observed in humans (84–86). Increasing evidence shows that these effects are reached after bioactivation of the polyphenols by the gut microbiota (87, 88). Even though plant polyphenols have protective effects on human health, especially in the bioactive form, there is still a possibility of adverse effects due to their complexity of action and potential inter-individual variability (89).

While not all types of microbes participating in polyphenol metabolism are yet known, it has recently been shown that *Bifidobacterium, Lactobacillus* sp., *Coriobacteriaceae, Clostridium* sp., *Bacteroides*, and *Saccharomyces* yeast, are involved in the process of converting polyphenols to equol, urolithins, and enterolignans (74, 88). The qualitative and quantitative proportions of urolithins and equol produced correlate positively with the effects of phytoestrogens (88). Other bacterial species, such as *Coriobacteriaceae* and *Eubacterium*, are responsible for different polyphenol transformations (88).

The interaction of polyphenols and gut microbiota is bidirectional (90, 91). The gut bacteria produce microbial metabolites from polyphenols, which in turn serve as prebiotics for the gut bacteria. These metabolites, particularly urolithins, promote the growth of *Lactobacillus* and *Bifidobacterium*(88).

### Vitamins

Gut microbiota are crucial for adequate vitamin levels in the human body. Menaquinone, folate, cobalamin, and riboflavin (ie: vitamins K, B9, and B2) are produced by gut microbes (25). Different bacteria have biosynthetic properties for different vitamins, such as *Bifidobacterium* for vitamins K, B_12_, biotin, folate, thiamine, *Bacillus subtilis* and *Escherichia coli* for riboflavin (92), and *Lactobacillus* for cobalamin and other B vitamins (93). The pathway analysis of the predicted metagenomes showed an enrichment of folate biosynthesis in vegans compared with omnivores (77).

### Isothiocyanates

Isothiocyanates are compounds from glucosinolates, mainly found in plants, like cruciferous vegetables. *Escherichia coli*, certain *Bacteroides*, some *Enterococcus, Lactobacillus agilis*, certain *Peptostreptococcus* spp. and *Bifidobacterium* spp. metabolize glucosinolates to isothiocyanates, secreting their own myrosinase enzyme (94). These metabolites have cytoprotective and anti-oxidative effects through regulation of gene expression relating toneoplastic, atherosclerotic, and neurodegenerative processes (25).

### Aryl-Hydrocarbon Receptor Ligands

Intestinal aryl-hydrocarbon receptor ligands are predominantly diet derived from plant food, specifically cruciferous vegetables. Through aryl-hydrocarbon receptors, the ligands act to promote intestinal immune function and gut homeostasis (95). Since aryl-hydrocarbon receptor ligands are gut microbiota-derived, any impairment to the gut microbiota, such as from a high-fat diet, can decrease aryl-hydrocarbon receptor ligands. In turn, this can cause gut inflammation and permeability and promote the development of metabolic syndrome, which can be improved by supplementation with a *Lactobacillus* strain (96). Additionally, a decrease in aryl-hydrocarbon receptors or ligands compromises the maintenance of intraepithelial lymphocytes and the control of the microbial load and composition, resulting in heightened immune activation and epithelial damage (95).

### Secondary Bile Acids and Coprostanol

A separate group of postbiotics are cholesterol metabolites. Several bacterial strains, isolated from intestine or feces, are described to convert dietary or synthesized *de novo* cholesterol into coprostanol (97, 98), which is poorly absorbed by the human intestine. Thus, serum cholesterol in host is reduced, which decreases the risk of cardiovascular diseases. On the other hand, bile acids synthetized from cholesterol are converted by microbiota into secondary bile acids, found in different tissues and in feces. It is believed secondary bile acids are involved in the equilibrium of health/disease (73, 97). For example, they are associated with inflammatory bowel disease, liver and colon cancer.

### Trimethylamine N-Oxide (TMAO)

Trimethylamine N-Oxide is a microbial metabolite believed to be associated with cardiovascular and neurological disorders. Carnitine and choline are the precursors of TMAO and are primarily found in foods of animal origin (eggs, beef, pork), with lower amounts found in beans and fish (99). Several microbial genera, like L-*Ruminococcus*, have been linked to the intake of animal proteins and fat and have been associated with TMAO levels (77). In general, meat intake appears to proliferate species of *Bacteroides, Alistipes, Ruminococcus, Clostridia*, and *Bilophila*, and decease *Bifidobacterium*. Higher TMAO levels have also been observed with red meat intake, increasing risk for cardiovascular disease and inflammatory bowel disease (54, 66). Vegetarians have a different gut microbiota composition than omnivores with a diminished capacity to produce trimethylamine (TMA), the precursor to TMAO. The plasma concentrations of TMAO appear to be similar in vegans and lacto-ovo-vegetarians (99, 100).

Lowering TMAO levels may be achieved through greater adherence to the Mediterranean diet, particularly a vegetarian one rich in fruits and vegetables (77, 100). Increased vegetable consumption reduces TMAO levels by reducing the enzymes responsible for converting TMA to TMAO and by remodeling the gut microbiota. The studies have shown TMAO production to decrease in vegetarians, which decreases their cardiovascular risk. To be objective, we have to mention a recent study, leaving a room for further analyses. Vegan fecal microbiota transplantation in metabolic syndrome patients resulted in significant changes in intestinal microbiota composition but failed to show changes in TMAO production. Authors explained that the 2-week follow-up was not a sufficient length of time to observe changes in TMAO production (101).

On average, twenty five percent of plasma metabolites are different between omnivores and vegans, suggesting a significant direct effect of diet on the host metabolome. No unique bacterial taxa have been significantly associated with individual metabolite levels after adjustment for multiple comparisons (102). These findings suggest that while inter-individual variability exists, dietary patterns significantly influence the microbial composition.

## Conclusion

Current research indicates that diet is the essential factor for human gut microbiota composition, what in its turn is crucial for metabolizing nutrients into active for the host postbiotics. Up to date knowledge suggests that a plant-based diet may be an effective way to promote a diverse ecosystem of beneficial microbes that support overall health. Nonetheless, due to the complexity and inter-individual differences, further research is required to fully characterize the interactions between diet, the microbiome, and health outcomes.

## Author Contributions

AT and IB contributed to conception and writing of the manuscript, ER, WY, JA, NB, and HK contributed and critically revised the manuscript. All authors approved the final manuscript.

### Conflict of Interest Statement

The authors declare that the research was conducted in the absence of any commercial or financial relationships that could be construed as a potential conflict of interest.

## Footnotes

^1^https://www.ars.usda.gov/ARSUserFiles/80400530/pdf/dbrief/12_fiber_intake_0910.pdf. (Accessed February 12, 2019)
